# Motion detection or time-lapse? A comparison of camera trap triggers for monitoring breeding taiga bean geese (*Anser fabalis fabalis*)

**DOI:** 10.1371/journal.pone.0340055

**Published:** 2026-01-07

**Authors:** Milaja Nykänen, Hannu Pöysä, Juho Matala, Mervi Kunnasranta

**Affiliations:** 1 Department of Environmental and Biological Sciences, University of Eastern Finland, Joensuu, Finland; 2 Natural Resources, Natural Resources Institute Finland (Luke), Joensuu, Finland; HUN-REN Centre for Ecological Research, HUNGARY

## Abstract

Game cameras have emerged over the recent years as an effective research tool for collecting various types of data on wild animals, and they are used increasingly also in avian studies. However, choosing the best method to collect data depends on the aim of the research and the characteristics of the target species and its habitat. Here, we compared the performance of two trigger methods of game cameras, passive infrared (PIR) motion sensor and time-lapse triggering, in capturing images of taiga bean goose (*Anser fabalis fabalis*) during two successive breeding seasons in peatlands across Finland in 2020–2021. While accounting for differences in camera effort (difference in the number of hours cameras using different trigger types were operational), we found daily capture probability (probability of at least one goose being present in photos during one day) associated with time-lapse to be marginally higher compared to motion triggered cameras. However, there was no difference in the daily number of geese between the two trigger modes. We also found the daily capture probability and detected number of geese to vary significantly between years, but this could be attributed to random inter-annual variation. In general, we find 15-minute interval time-lapse to be a more suitable method compared to motion triggered cameras to study seasonally elusive ground dwelling birds like the taiga bean goose due to fewer required visits to camera sites and thus less disturbance caused to the birds during the sensitive breeding period. However, using cameras with both trigger types side-by-side would likely lead to best overall capture probability, as indicated by the higher percentage of detection positive time periods when goose detections from both camera types were combined, compared to the percentage derived for either of the trigger types alone.

## Introduction

Camera trapping has become a powerful research tool for collecting data on wildlife because it can be carried out at a relatively low cost compared to other survey or monitoring methods that would require extended human presence in the study area. Moreover, its non-invasive nature in data collection enables the monitoring of elusive species, also in remote locations and during long periods of time [1–3]. This approach has traditionally been used to gather information on various population metrics of large terrestrial mammals such as occurrence [4] and abundance [5,6], and behaviour patterns [7,8], but nowadays it is used increasingly for birds [2,9–11]. It is especially useful in the study of ground dwelling birds, where game cameras have been recently used to describe, for example, activity patterns [12], foraging [13], habitat use [14,15], abundance [9], predation pressure [16], nest productivity and chick survival [17].

Despite the potential of game cameras as an effective research tool in a range of applications [18], they may also have several limitations associated with them, such as variability in camera performance (*e.g.*, issues with time-keeping) or challenges in sampling design [6,19–21]. One key aspect in camera performance is the trigger mode: in *motion sensor* triggering a passive infrared (PIR) sensor triggers the camera to capture an image, whereas in *time-lapse* triggering the camera is programmed to take images at a predefined time interval. The latter represents an application of the instantaneous sampling recording rule, which is a type of scan sampling, because it records the “state” of the observed subject at discrete points in time [22]. Problems may occur, if the camera produces false triggers leading to vast amounts of blank or empty images and therefore drains batteries and fills memory card space. This is particularly the case with motion sensor triggering when false triggers are caused by, for example, swinging vegetation or fluctuation in the light levels. Although most game cameras allow users to adjust the sensitivity when using motion triggers which can help alleviate some of the false triggering, there may be a downside to this as detections of target species can be missed entirely if the trigger sensitivity is set too low. On the other hand, time-lapse triggering can also lead to a large number of empty images for a species that is harder to detect or only intermittently present (for example at a den entrance), in which case the vast majority of images may be empty, and the species may be missed entirely. All these scenarios may cause extra work for researchers and/or lead to bias in the results. The goal is to find the most suitable method for a specific species and environment that will achieve a good balance between sufficient number of images of the target species and the amount of manual processing work.

Here, we compare the performance of motion triggered and time-lapse camera trap settings in gathering occurrence and count data on the bean goose (*Anser fabalis*) in its breeding areas in Finland. The bean goose is a sporadic breeder in remote and hard-to-access habitats in the arctic and boreal zones from Fennoscandia to Western and Eastern Siberia [23–27]. The Western Palearctic population of the species, belonging to the subspecies taiga bean goose (*A. f. fabalis*), has declined in previous decades [28,29] with the conservation status of the subspecies considered as Vulnerable in Finland [30]. However, during the past 10–15 years the population has shown some signs of recovery [31]. While efforts have been put into increasing the accuracy of methods used to estimate taiga been goose numbers in the nonbreeding season [32], monitoring its numbers in the breeding season remains a challenging obstacle to efficient conservation of the subspecies. During the breeding season taiga bean goose is elusive and difficult to detect, especially when nesting and during early brood rearing, and any survey method involving disturbance caused by the presence of human observers could further reduce the detectability of the species [33].

The main objective of this study was to gain a better understanding of game camera trap trigger performance and to find the most cost-effective data collection procedure for bean geese during the breeding season. As the geese are not distinctively marked and cannot be individually identified, the purpose of this study was not to derive an abundance estimate for the subspecies due to possible double-counting of the same individuals. Instead, our aim was to find out which trigger type (motion sensor or time-lapse) captures greater counts of detected geese or is associated with higher daily capture probability (defined here as probability of at least one goose being present in photos during one day), the latter being critical in providing occurrence data. Finding the best available method would help towards developing an effective monitoring method for the taiga bean goose population during the breeding season.

## Materials and methods

### Taiga bean goose

Several natural history characteristics of the taiga bean goose make the species difficult to monitor during the breeding season with conventional methods but particularly suitable for camera trapping studies (see also Introduction). The taiga bean goose is a large grey goose, about 66‒84 cm tall [24]. In contrast with other large goose species, many of which belong to the tribe Anserini and nest colonially [24], detectability of the taiga bean goose is low in the breeding areas. They nest solitarily and breeding pairs are very difficult to detect in remote peatlands due to their elusive behaviour during nesting and early brood stages. Taiga bean goose nests are well dispersed and can be situated in open habitats typically within 2 km of open water as well as in wooded habitats such as dense coniferous forests around mires. While nesting density in the current study area is not known, it is generally low throughout the subspecies range [24]. Home ranges of breeding pairs are large, even up to 1000‒2000 hectares [33]. However, during the brood rearing and moulting period in summer, broods can be seen around water bodies of open and wooded mires, where camera traps can be deployed.

### Study area and camera placement

**Fig 1:**
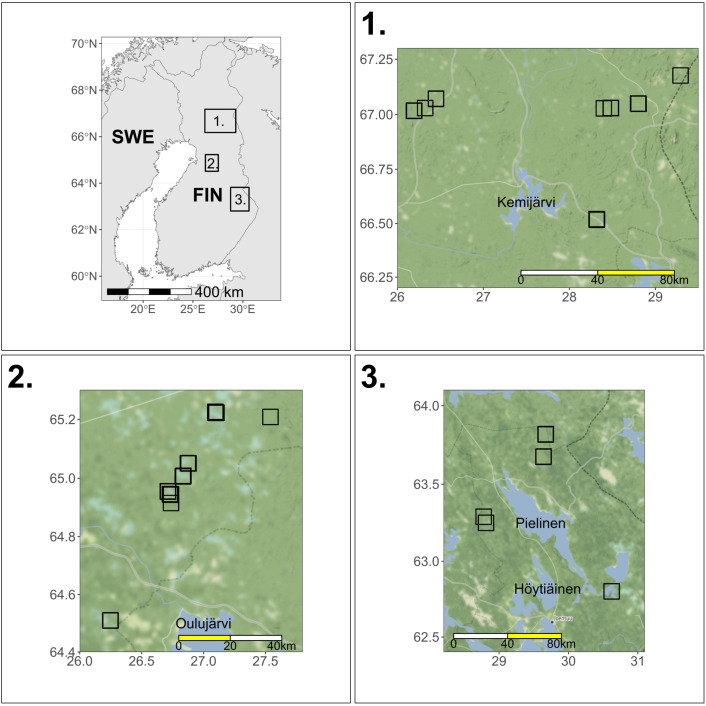
Study areas. Study locations (black rectangles) containing peatland ponds where camera traps were deployed in 2020 and 2021, in the provinces of Lapland (1), Northern Ostrobothnia (2), and North Karelia (3). Note that due to map scale, individual peatland ponds (see an example of one in Fig 2) are not visible on the map. The maps were drawn under CC BY 3.0 (data by OpenStreetMap, under ODbL). The inset map was drawn using Natural Earth map data.

**Fig 2:**
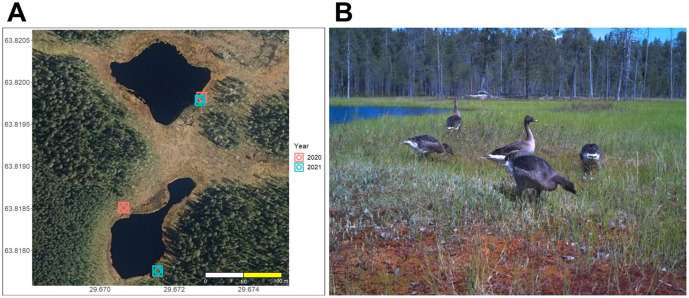
a) The camera placement in one of the study sites, a peatland pond Pirttilammit in North Karelia. The rectangles denote motion triggered and circles time-lapse cameras, with the years marked in different colour, and b) an example of an image where taiga bean geese were captured with a PIR sensor triggered camera in Pirttilammit, North Karelia, in 2021.

The study regions covered 19 peatlands across Finland in 2020 and 2021 (Fig 1), all known to have bean geese present during previous years [12]. The peatlands are located in the provinces of North Karelia (N = 5), Northern Ostrobothnia (N = 8) and Lapland (N = 6). Fifty-six and fifty-two game cameras (28 and 26 of each trigger type) were deployed in 2020 and 2021, respectively, for the duration of the breeding period from the beginning of May to September, covering the season when the geese were nesting, caring for the offspring, and moulting (Tables S1 and S2, S1 Appendix). Study sites (peatland ponds) were divided into 6.25 ha grids (250 m × 250 m) and one or two grids, depending on size of the peatland, were randomly selected for camera trap monitoring (see [12]). In 2020, there were six ponds with a single grid and 11 ponds with two grids, and in 2021, there were nine single-grid and nine two-grid ponds (Tables S1 and S2 in S1 Appendix). The study sites were otherwise the same during both years, except one peatland pond in Lapland in 2020 (Vasa-Aapa, 67.17670N, 29.29121E) had to be replaced by another (Suikeloaapa, 67.0721N, 26.449967E) in 2021 due to logistical difficulties in setting the cameras. In each study site, 1–2 camera pairs (with a single camera type, Uovision UV785 Full HD 12 MP), consisting of one motion activated camera and one time-lapse camera, were placed in the grids (see example in Fig 2a). The same grids were not necessarily available during both years due to fluctuations in water level, and each year the placement within grids was chosen based on high usage by geese observed during the first visit. Cameras set to motion activated triggering were programmed to capture two still images with a 10 s delay between triggers, and cameras set on time-lapse cameras were programmed to take two still images every 15 minutes. All cameras were set to record images 24 hours a day as the camera model is equipped with a “no-glow” black LED that enables photographing objects that are within 15 m range during the hours of darkness. Memory card size was 16GB, alkaline AA-batteries were used, and image resolution was 8MP in both trigger types. In motion sensor triggered cameras, sensitivity was set “low”, and PIR interval was 10 s. Further, according to the camera manual, the PIR detection range for the motion sensor mode is 16 m for a human-sized target. Cameras in a camera pair were attached on top of each other (time-lapse above the motion triggered camera) to a tree or wooden pole at a height of 1 m above the ground. The placement of the cameras was right on top of each other to ensure they captured the same view. Cameras were typically visited (during daylight hours) once or twice during the study period to replace the memory cards and batteries, if needed. In case geese were observed during these visits, replacing of batteries and memory cards was delayed in order to avoid disturbance. After each field season, adult taiga bean geese and goslings were detected and counted manually from the images (Fig 2b), and totalled for 12 two-hour time periods (*i.e.*, 00:00–01:59, 02:00–03:59…) for each ordinal (Julian) day. The count was then summarized as the total count of geese (both adults and goslings added together) for each camera day.

No permits concerning animal welfare/ethics for bean goose camera trapping were required as birds were not intentionally approached, and remote camera traps are considered to be a non-invasive study method. Camera traps were set with landowners’ permission on private land and on state-owned areas with Metsähallitus permit (permit number MH1145/2018).

### Statistical analyses

A generalized linear mixed model (GLMM) was used to investigate whether there was a difference in the detected daily count of taiga bean geese (adults and goslings added together) captured by cameras triggered by motion sensor vs cameras set to time-lapse. The GLMM was run in R [34] using the package glmmTMB [35] with the factors ‘trigger type’ (motion sensor and time-lapse) and ‘year’ (2020 and 2021) as fixed factors and with the factor ‘site’ (19 individual peatland ponds) included as a random intercept in the model. The possible effects of other variables were not explored in this study, as a previous study already investigated the relationship between taiga bean goose activity and seasonality, time of day and some environmental variables [12]. We acknowledge that leaving out sources of variability (*e.g*., temporal or habitat-related) from the models may lead to some overdispersion; however, as the game cameras operating with two trigger types were paired, this should not impact the results or their interpretation. The varying camera effort, resulting from the different number of cameras deployed on the study sites and the amount of time that the cameras were recording over the study period, was accounted for by including an offset-term (approximated as a continuous variable) in the model that was calculated by totalling the number of 2 h time periods of recording per trigger type for each day. The effort was thus taking a value between 1 (one camera per site operating during only one 2 h time period) and 24 (two cameras at a site operating throughout all of the twelve 2 h time periods). Due to the data including a large number of zero values (most days had zero goose counts), we ran different candidate models with a Tweedie and Poisson distributions (both with log-link function), with and without accounting for the zero-inflation (See Table 1). Further, to account for possible overdispersion, we ran two types of negative binomial distributions, NB1 (variance = μ(1 + ϕ), where µ is the mean (or expected count) and ϕ is the dispersion parameter of the negative binomial distribution) and NB2 parameterizations (variance = µ(1 + µ/ϕ)) [36,37], also with and without accounting for zero-inflation.

**Table 1:** Summary of candidate count models. Summary of candidate models ran to investigate the effect of trigger type (time-lapse or motion sensor) on the daily count of taiga bean geese captured on game camera traps. ‘Effort’ means camera effort, which was calculated by totalling the number of 2 h time periods of recording per trigger type for each day. NB stands for negative binomial with two different parameterizations, NB1 (variance = μ(1 + ϕ), where µ is the mean (or expected count) and ϕ is the dispersion parameter of the negative binomial distribution) and NB2 (variance = µ(1 + µ/ϕ)) [36,37]. Models are ranked by AICc from lowest (AICc-top) to highest. The table includes the number of estimated parameters, *K*; both AICc and ΔAICc which is AICc minus the lowest AICc and the negative log-likelihood, *L.*

Model formulation	Distribution and type	*K*	*L*	AICc	ΔAICc
Goose count ~ Year + Trigger type + offset(log(effort)) + (1|Site)^a^	NB1 + zero-inflation	6	−4151.4	8314.7	0.0
Goose count ~ Year * Trigger type + offset(log(effort)) + (1|Site)^a^	NB1 + zero-inflation	7	−4150.8	8315.6	0.9
Goose count ~ Trigger type + offset(log(effort)) + (1|Site)^a^	NB1 + zero-inflation	5	−4160.6	8331.2	16.5
Goose count ~ Year + Trigger type + offset(log(effort)) + (1|Site)^a^	Tweedie	6	−4164.0	8339.9	25.2
Goose count ~ Year * Trigger type + offset(log(effort)) + (1|Site)^a^	Tweedie	7	−4163.9	8341.7	27.0
Goose count ~ Year + Trigger type + offset(log(effort)) + (1|Site)^a^	Tweedie + zero-inflation	7	−4164.0	8341.9	27.2
Goose count ~ Year * Trigger type + offset(log(effort)) + (1|Site)^a^	Tweedie + zero-inflation	8	−4163.9	8343.7	29.0
Goose count ~ Trigger type + offset(log(effort)) + (1|Site)^a^	Tweedie	5	−4176.4	8362.9	48.2
Goose count ~ Trigger type + offset(log(effort)) + (1|Site)^a^	Tweedie + zero-inflation	6	−4176.4	8364.9	50.2
Goose count ~ Year + Trigger type + offset(log(effort)) + (1|Site)^a^	NB1	5	−4210.9	8431.7	117.0
Goose count ~ Year * Trigger type + offset(log(effort)) + (1|Site)^a^	NB1	6	−4210.6	8433.1	118.4
Goose count ~ Trigger type + offset(log(effort)) + (1|Site)^a^	NB1	4	−4225.2	8458.4	143.7
Goose count ~ Year + Trigger type + offset(log(effort)) + (1|Site)^a^	NB2 + zero-inflation	6	−4225.0	8462.1	147.4
Goose count ~ Year * Trigger type + offset(log(effort)) + (1|Site)^a^	NB2 + zero-inflation	7	−4224.1	8462.3	147.6
Goose count ~ Trigger type + offset(log(effort)) + (1|Site)^a^	NB2 + zero-inflation	5	−4229.3	8468.6	153.9
Goose count ~ Year + Trigger type + offset(log(effort)) + (1|Site)^a^	NB2	5	−4286.5	8583.0	268.2
Goose count ~ Year * Trigger type + offset(log(effort)) + (1|Site)^a^	NB2	6	−4286.3	8584.5	269.8
Goose count ~ Trigger type + offset(log(effort)) + (1|Site)^a^	NB2	4	−4289.9	8587.7	273.0
Goose count ~ Year * Trigger type + offset(log(effort)) + (1|Site)^a^	Poisson + zero-inflation	6	−6168.0	12343.9	3870.5
Goose count ~ Year + Trigger type + offset(log(effort)) + (1|Site)	Poisson + zero-inflation	5	−6092.2	12194.4	3879.7
Goose count ~ Trigger type + offset(log(effort)) + (1|Site)^a^	Poisson + zero-inflation	4	−6168.0	12343.9	4029.2
Goose count ~ Year * Trigger type + offset(log(effort)) + (1|Site)^a^	Poisson	5	−15168.9	30347.9	22033.2
Goose count ~ Year + Trigger type + offset(log(effort)) + (1|Site)^a^	Poisson	4	−15170.5	30349.0	22034.3
Goose count ~ Trigger type + offset(log(effort)) + (1|Site)^a^	Poisson	3	−15389.2	30784.4	22469.7

^a^ random intercept of ‘site’.

We also ran a set of logistic GLMMs (see Table 2) using the R-package glmmTMB [35] to investigate whether the camera trigger type affects the daily capture probability (probability of at least one goose being present in photos during one day) of taiga bean geese. Since it was not possible to include the offset-term in this type of model, we added the camera effort as a continuous covariate in the models. Other covariates were included as fixed (‘trigger type’ and ‘year’) or random (‘site’) factors the same way as in the count models. In addition, we compared the detections (goose/geese present in images) within each camera pair (motion sensor and time-lapse camera placed in the same location) for those 2 h time periods when both cameras were operational and calculated the failure rates of each camera type compared to one another. In other words, we considered it a failure for the motion triggered camera if a camera operating on a time-lapse setting captured at least one goose over a 2 h period but the adjacent motion triggered camera did not record any geese during the same time period, and vice versa.

**Table 2:** Summary of candidate models for capture probability. Summary of candidate models ran to investigate the effect of trigger type on the capture probability (probability of at least one goose being present in photos during one day) of taiga bean geese captured on game camera traps. ‘Effort’ means camera effort, which was calculated by totalling the number of 2 h time periods of recording per trigger type for each day. Models are ranked by AICc from lowest (AICc-top) to highest. The table includes the number of estimated parameters, *K*; both AICc and ΔAICc which is AIC, minus the lowest AICc and the negative log-likelihood, *L.*

Model formulation	Distribution and type	*K*	*L*	AICc	ΔAICc
Goose presence ~ Year + Trigger type + effort + (1|Site)^a^	Binomial (logit-link)	5	−1840.6	3691.1	0.0
Goose presence ~ Year + Trigger type * effort + (1|Site)^a^	Binomial (logit-link)	6	−1840.0	3692.0	0.9
Goose presence ~ Year * Trigger type * effort + (1|Site)^a^	Binomial (logit-link)	9	−1837.5	3693.1	1.9

^a^ random intercept of ‘site’.

We then compared model fits using Akaike’s Information Criterion (AIC) values to determine the best fitting models. The goodness of fit of the models was assessed and verified by creating scaled quantile residual plots via simulation using the R-package DHARMa [38].

## Results

Median start and end dates of camera deployment were 16th of June and 20th of August in 2020 and 2nd of June and 16th of August in 2021 (Tables S1 and S2, S1 Appendix). The mean duration of deployment of motion sensor and time-lapse triggered cameras in 2020 was 67 days (with standard deviation (SD) of 22) and 55 days (SD = 24), respectively. In 2021, the mean duration was 68 days (SD = 27) for motion triggered cameras and 60 days (SD = 29) for time-lapse cameras. Altogether, the motion triggered cameras captured 24,596 and 45,387 images and the time-lapse cameras 134,979 and 134,768 images in 2020 and 2021, respectively (Tables S1 and S2, S1 Appendix). Geese were present in images captured with motion triggered cameras in 631 out of 43,808 2-hour long time periods (0.8%) when cameras were recording, whereas the number of detection positive time periods was 836 out of 37,064 with cameras set to time-lapse (2.3%). At least one goose was detected with either camera type during 1,226 time periods out of 49,224 (2.5%).

**Fig 3:**
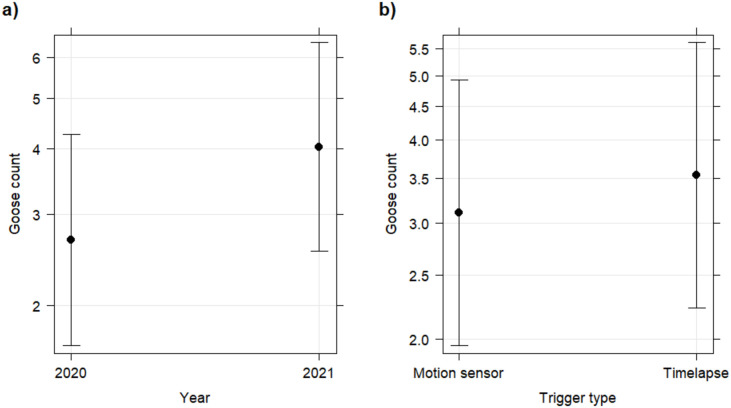
Conditional effects (on a response scale) of a) year and b) trigger type on the total number of taiga bean geese captured per day (goose count) with game camera traps on peatland ponds in Finland between 2020 and 2021. The difference was significant between years (p < 0.001) but not significant between trigger types (p = 0.129). Points represent the mean and whiskers the 95% confidence interval.

The best fitting count model had a negative binomial distribution (NB1 parameterization) and it accounted for zero-inflation (zero-inflation p < 0.001, Table 1). It included the fixed factors ‘trigger type’ and ‘year’ without their interaction and the random intercept of ‘site’ (random effect variance (σ^2^) of 0.906). The daily taiga bean goose count was significantly higher in 2021 compared to 2020 (p < 0.001, Fig 3); however, the camera trigger type had no significant effect on the detected count of geese (p = 0.129).

**Fig 4:**
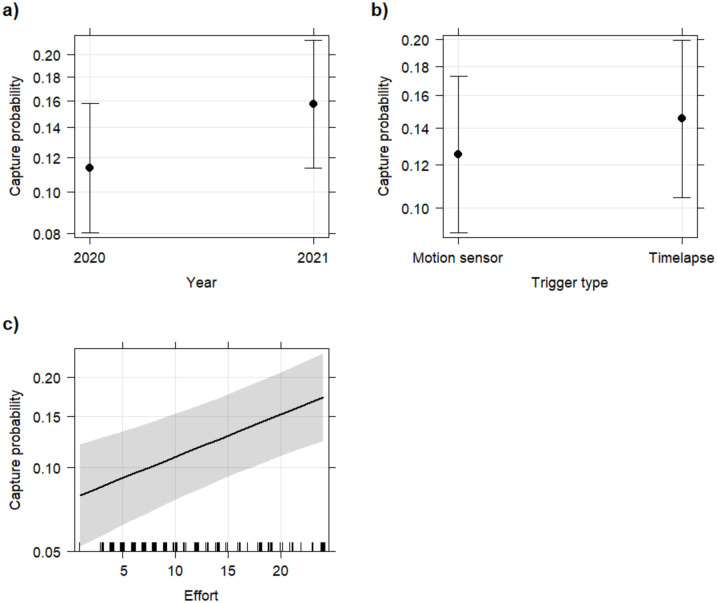
Conditional effects (on a response scale) of a) year, b) trigger type, and c) camera effort on the daily capture probability of taiga bean geese (probability of at least one goose being present in photos during one day) photographed with game camera traps. Camera effort is the number of 2 h time periods of recording per trigger type for each day. The difference was significant between years (p < 0.001) and marginally significant between trigger types (p = 0.049). The amount of camera effort had a significant positive effect on the daily capture probability (p < 0.001). Points represent the mean and whiskers the 95% confidence interval.

As the AIC values were within <2 of each other, the best fitting logistic model on goose capture probability (probability of at least one goose being presence present in photos during one day) was selected based on parsimony and it included the fixed terms ‘trigger type’, ‘year’ and ‘effort’ without interactions and the random intercept of ‘site’ (random effect σ^2^ = 0.622, Table 2). The capture probability increased significantly with increasing camera effort (p < 0.001) and was significantly higher in the year 2021 (p < 0.001) and marginally significantly higher (p = 0.049) with game cameras set to time-lapse (Fig 4). When including only the time periods that both camera types in each pair were operational, time-lapse cameras recorded 721 goose presences, with motion triggered cameras failing to detect ~67% of them. On the other hand, motion triggered cameras recorded 478 images with at least one goose present while the time-lapse cameras missed ~50% of those occasions.

## Discussion

Time-lapse cameras may allow the collation of more standardized data than motion triggered cameras and potentially reduce the number of empty images produced by false triggering. Our study shows that, even though no difference was found in the daily count of detected geese captured with the two trigger types, the overall daily capture probability of taiga bean geese was marginally higher with cameras set to time-lapse capturing images with a 15-minute interval compared to motion triggered cameras. Further, when comparing the two trigger types within the same camera pair, the time-lapse cameras had much lower failure rate to detect geese (50%) compared to motion triggered cameras (67%).

The fact that there were no differences in goose count between the two trigger types but that time-lapse cameras performed slightly better when considering capture probability and failure rate could at least partly be explained by the motion triggered cameras failing to capture the more distant animals, especially when they are alone, whereas the time-lapse method captures both nearby and distant animals. This makes positioning of the camera traps operating on a time-lapse setting less critical as animals can be detected even if they do not use their exact assumed route or site. Indeed, in order for the animal to be captured with the motion trigger mode, the PIR sensor must detect motion in the trigger area while the animal is within the camera’s viewable area [39]. In most game camera models (such as the one used in this study) it is possible to adjust the PIR-sensitivity higher. Higher PIR-sensitivity increases the detection range by enabling the sensor to pick up on small fluctuations in infrared light further in front of and at the sides of the camera. However, it also increases the risk of false detection or can lead to missed detections if the sensor is triggered while the target is moving from behind the camera to the front and is still outside the viewable area. Both cases will lead to a larger number of empty images. It is important to note that we tested only one commonly used model of game cameras from a single manufacturer in this study (using only the low PIR-sensitivity), and it is possible that other camera models with a longer PIR sensor detection range would achieve better capture probability (as we define it).

Our study on taiga bean geese in their breeding areas suggests that time-lapse triggering mode operating on a 15-minute interval performs as well or even slightly better compared to motion trigger mode (on a low sensitivity setting) when collecting data on the occurrence of geese. Moreover, even though the camera pairs in each site were visited in this study at the same time due to logistical reasons, using 15-minute time-lapse interval trigger would, in theory, reduce the need to visit the camera sites for changing batteries and memory cards, hence minimising the disturbance during the sensitive breeding period of the geese. However, our study was limited to just one time-lapse interval. While having a shorter, for example 5-minute, interval between images would likely result in a higher capture probability and larger goose counts, it would also lead to more images and greater manual processing time and possibly to faster drainage of batteries, filling of memory card space and thus also to increased number of visits to the field sites. This emphasizes the need to find the most suitable trigger interval that captures the greatest number of images of the target animal while keeping the number of empty images and image processing time manageable. Also, recent developments in batteries (*e.g*., the possibility to attach solar panels to charge the batteries) and image storage (*e.g*., larger size memory cards) could offer alternative ways to minimize the need to physically revisit cameras and, thus, disturbance. An important advantage of using time-lapse photography is that it produces standardized data (i.e., photos are taken at regular fixed intervals) on the wider site use of taiga bean geese in their breeding areas compared to motion triggered cameras due to failure of the latter to capture far-away targets. However, using cameras with both trigger types side-by-side would likely lead to best overall capture probability, as indicated by the higher percentage of detection positive time periods when goose detections from both camera types were combined (2.5%), compared to the percentage derived for either time-lapse (2.3%) or motion activated triggering (0.8%) alone.

Choosing the triggering mode depends on the research objectives and characteristics of the target species and its habitat. As our study shows, time-lapse may be more suitable, for example, in studies involving presence/absence data -based habitat monitoring when it is important to distinguish between used and unused sites. Motion trigger mode, on the other hand, may be more useful in cases where the density of the animals or their probability to visit camera sites is low, as it is possible that the time-lapse method may fail to detect the target species altogether [39]. Further, it may be a better option in studies on animal movement or behaviour, or when the area to be monitored is fairly small or the animals are known to use a predetermined route, for example at a den entrance. For example, motion triggered cameras have been shown to capture a higher proportion of crossing events of small and large mammals than cameras operating on time-lapse in a study monitoring wildlife road underpass usage, although the best results were obtained by using a combination of motion triggering and time-lapse [40].

Similar to our study, Leorna and Brinkman [41] recorded higher proportions of images containing at least one caribou (*Rangifer tarandus*) using time-lapse triggered cameras with the interval between images set between 5 and 120 min compared to motion triggered cameras. However, the authors deemed the motion sensor setting more suitable for their study subject as the capture rate of images collected (defined by the authors as the proportion of images containing ≥1 caribou out of all images collected) with motion sensor was more than 11-times greater than time-lapse due to the vast amount of images collected. Also in the present study, the number of photographs collected over the 2-year study period with the time-lapse method was nearly four times greater compared to motion sensor triggered cameras, and this naturally led to longer image processing time. However, we believe that the higher capture probability (as we define it), combined with the predictability in battery usage and image storage capacity, outweighed the increased cost of manual processing.

Camera trapping has gained popularity in wildlife studies over the recent years due to its efficiency to collect image data without the need to dedicate numerous hours of researchers’ time present on the study site. At the same time, large volumes of images collected with this method continue to be one of the challenges in the subsequent data management and analysis. For example, a single camera operating on time-lapse with a 15-minute interval, a setting used in this study, outputs 96 photos in a 24-hour period, and accumulates a dataset containing more than a thousand images over a study period longer than ten weeks. On the other hand, motion triggering may produce thousands of “empty” images in a short time period that still need to be checked for the presence of the target species. In total, our two-year study period produced nearly 350,000 images, which all were gone through manually, forming the most labour intensive and costly part of the study.

In order to reduce the photograph processing time, machine learning techniques (automatic identification algorithms) and/or a citizen science approach (*e.g*., [42–44]) have the potential to become some of the most important innovations in the cost-effective identification and counting of animals from large camera trap datasets in future studies. Indeed, Microsoft AI’s MegaDetector V4.1 [45], a type of computer vision model, performed very well compared to manual human review with over 90% correctly labelled images when only motion sensor captured images were considered [46]. However, its performance in identifying targets from time-lapse images was much poorer with only ~60% correctly labelled images of animals compared to the human eye, hypothesized by the authors to be an artefact of the much smaller minimum detection size limit (and thus much greater detection distance) of human observers compared to MegaDetector. Thus, the performance of any computer vision method should be thoroughly tested and its suitability for the study system assessed before adopting it to monitoring schemes.

We found a difference in the daily count of detected geese and in their capture probability between the two study years. Unfortunately, because standardized monitoring data of the annual numbers of the taiga bean goose in the breeding areas are not available, it is not possible to say if the between-year difference reflects a real difference in breeding numbers or changes to their breeding distribution. Moreover, the fact that cameras were deployed at study sites almost two weeks earlier in 2021 compared to 2020 (start of June in 2021 and mid-June in 2020, measured in median deployment dates) could have contributed to the observed higher capture probability of taiga bean geese in 2021. This is because non-breeding birds (individuals that failed or skipped breeding) from breeding sites in Finland migrate elsewhere later in June to moult, leaving only the successful breeders at the breeding sites [47]. It is possible that the earlier game camera deployment in 2021 led to capturing both breeding and non-breeding geese whereas in 2020 mostly the successful breeders were captured on cameras. Nevertheless, the findings of this study and those of Nykänen et al. [12], together covering data from four successive breeding seasons (2018‒2021), suggest that game cameras are a feasible cost-effective method for monitoring taiga bean goose during the breeding season. First, the data acquired in this study would be extremely difficult to obtain using human observers as the species is timid during breeding season [48] and breeding in remote hard-to-access areas. Second, with a careful consideration of the timing when game cameras are deployed or limiting data analysis to a specific time period, *i.e*., having the monitoring/data period to cover only later part of the summer to capture only the successful breeders, they could reveal between-year differences in the number of breeding pairs and their offspring at monitored sites. While information on the number of different individuals (actual abundance) is not possible to derive from camera traps without marked individuals, the data from camera traps can be used to produce abundance indices for monitoring purposes like it has been done with other ground-dwelling birds [9] or, with using snow tracks, for mammals [49,50]. Third, game cameras set at fixed sites are a cost-effective method to gather highly comparable long-term data from remote breeding areas that are difficult to access and cover using other survey methods (S1-S2 File). And finally, non-expert citizens who live locally in the area could be engaged in the monitoring by deploying cameras in remote locations, making it possible to cover a large number of potentially important breeding sites. Our findings concerning taiga bean goose thus echo recent suggestions that camera traps can be used for monitoring wetland birds in boreal breeding areas [51].

## Supporting information

S1 File R-script used to analyse the taiga bean goose data. (TXT)

S2 File Taiga bean goose data. (CSV)

S1 Appendix Containing S1 and S2 Tables. (DOCX)
