# Supplementary material for: Motion detection or time-lapse? A comparison of camera trap triggers for monitoring breeding taiga bean geese (Anser fabalis fabalis)
Source: PLoS One. 2026 Jan 7;21(1):e0340055. doi: 10.1371/journal.pone.0340055 (PMC12779131; doi:10.1371/journal.pone.0340055)
Supplement: S1 Appendix — (DOCX) [file pone.0340055.s003.docx]

**Motion detection or time lapse? A comparison of camera trap triggers for monitoring breeding taiga bean geese (*Anser fabalis fabalis*)**

**Appendix S1**

Milaja Nykänen, Hannu Pöysä, Juho Matala, Mervi Kunnasranta

**Table S1. Details of game cameras deployed in 2020.**

| Year | Province | Site | Pair | Camera | Trigger | Start date | End date | Days | Images |
| --- | --- | --- | --- | --- | --- | --- | --- | --- | --- |
| 2020 | L | Vasa-Aapa | 1 | 83 | Motion sensor | 10.6.2020 | 25.8.2020 | 76 | 461 |
| 2020 | L | Vasa-Aapa | 1 | 29 | Time-lapse | 10.6.2020 | 28.7.2020 | 48 | 4512 |
| 2020 | L | Kaita-Aapa | 2 | 25 | Motion sensor | 10.6.2020 | 7.9.2020 | 89 | 373 |
| 2020 | L | Kaita-Aapa | 2 | 72 | Time-lapse | 10.6.2020 | 7.9.2020 | 89 | 7002 |
| 2020 | L | Kaita-Aapa | 3 | 70 | Motion sensor | 22.6.2020 | 7.9.2020 | 77 | 100 |
| 2020 | L | Kaita-Aapa | 3 | 76 | Time-lapse | 10.6.2020 | 7.9.2020 | 89 | 4556 |
| 2020 | L | Sourulampi | 4 | 51 | Motion sensor | 10.6.2020 | 7.9.2020 | 89 | 293 |
| 2020 | L | Sourulampi | 4 | 85 | Time-lapse | 10.6.2020 | 14.6.2020 | 3 | 359 |
| 2020 | L | Sourulampi | 5 | 53 | Motion sensor | 10.6.2020 | 7.9.2020 | 89 | 2351 |
| 2020 | L | Sourulampi | 5 | 54 | Time-lapse | 10.6.2020 | 6.8.2020 | 56 | 5217 |
| 2020 | L | Malja-Aapa | 6 | 77 | Motion sensor | 9.6.2020 | 8.9.2020 | 91 | 770 |
| 2020 | L | Malja-Aapa | 6 | 75 | Time-lapse | 9.6.2020 | 25.7.2020 | 46 | 4096 |
| 2020 | L | Malja-Aapa | 7 | 33 | Motion sensor | 8.6.2020 | 8.9.2020 | 92 | 123 |
| 2020 | L | Malja-Aapa | 7 | 52 | Time-lapse | 9.6.2020 | 6.8.2020 | 58 | 5346 |
| 2020 | NK | Lehmilampi | 8 | 44 | Motion sensor | 9.6.2020 | 25.6.2020 | 16 | 3957 |
| 2020 | NK | Lehmilampi | 8 | 71 | Time-lapse | 9.6.2020 | 11.6.2020 | 2 | 175 |
| 2020 | NK | Tavilampi | 9 | 67 | Motion sensor | 24.6.2020 | 15.9.2020 | 83 | 584 |
| 2020 | NK | Tavilampi | 9 | 56 | Time-lapse | 24.6.2020 | 24.8.2020 | 61 | 5717 |
| 2020 | NK | Teerilampi | 10 | 38 | Motion sensor | 24.6.2020 | 15.9.2020 | 83 | 1726 |
| 2020 | NK | Teerilampi | 10 | 36 | Time-lapse | 24.6.2020 | 15.9.2020 | 83 | 7785 |
| 2020 | NK | Rääkälelammit | 11 | 22 | Motion sensor | 25.6.2020 | 15.9.2020 | 82 | 154 |
| 2020 | NK | Rääkälelammit | 11 | 11 | Time-lapse | 25.6.2020 | 3.8.2020 | 39 | 3632 |
| 2020 | NK | Rääkälelammit | 12 | 49 | Motion sensor | 25.6.2020 | 16.8.2020 | 52 | 729 |
| 2020 | NK | Rääkälelammit | 12 | 32 | Time-lapse | 25.6.2020 | 15.9.2020 | 82 | 7212 |
| 2020 | NK | Pirttilammit | 13 | 8 | Motion sensor | 25.6.2020 | 12.9.2020 | 79 | 86 |
| 2020 | NK | Pirttilammit | 13 | 7 | Time-lapse | 25.6.2020 | 12.9.2020 | 79 | 6389 |
| 2020 | NK | Pirttilammit | 14 | 6 | Motion sensor | 25.6.2020 | 12.9.2020 | 79 | 600 |
| 2020 | NK | Pirttilammit | 14 | 5 | Time-lapse | 25.6.2020 | 31.8.2020 | 67 | 6293 |
| 2020 | NO | Pieni Mustikkalampi | 15 | 50 | Motion sensor | 15.6.2020 | 20.8.2020 | 66 | 493 |
| 2020 | NO | Pieni Mustikkalampi | 15 | 12 | Time-lapse | 15.6.2020 | 20.8.2020 | 66 | 6158 |
| 2020 | NO | Pieni Mustikkalampi | 16 | 55 | Motion sensor | 15.6.2020 | 20.8.2020 | 66 | 1627 |
| 2020 | NO | Pieni Mustikkalampi | 16 | 82 | Time-lapse | 15.6.2020 | 20.8.2020 | 66 | 6098 |
| 2020 | NO | Mustikkalampi | 17 | 15 | Motion sensor | 15.6.2020 | 20.8.2020 | 66 | 401 |
| 2020 | NO | Mustikkalampi | 17 | 17 | Time-lapse | 15.6.2020 | 20.7.2020 | 35 | 3233 |
| 2020 | NO | Mustikkalampi | 18 | 13 | Motion sensor | 15.6.2020 | 20.8.2020 | 66 | 995 |
| 2020 | NO | Mustikkalampi | 18 | 84 | Time-lapse | 15.6.2020 | 20.8.2020 | 66 | 5307 |
| 2020 | NO | Kalhulampi | 19 | 74 | Motion sensor | 16.6.2020 | 17.6.2020 | 1 | 13 |
| 2020 | NO | Kalhulampi | 19 | 66 | Time-lapse | 16.6.2020 | 20.8.2020 | 65 | 5833 |
| 2020 | NO | Kalhulampi | 20 | 57 | Motion sensor | 16.6.2020 | 20.8.2020 | 65 | 337 |
| 2020 | NO | Kalhulampi | 20 | 88 | Time-lapse | 16.6.2020 | 20.8.2020 | 65 | 6035 |
| 2020 | NO | Särkilampi | 21 | 39 | Motion sensor | 16.6.2020 | 27.7.2020 | 41 | 865 |
| 2020 | NO | Särkilampi | 21 | 87 | Time-lapse | 16.6.2020 | 21.8.2020 | 66 | 6155 |
| 2020 | NO | Särkilampi | 22 | 80 | Motion sensor | 16.6.2020 | 21.8.2020 | 66 | 921 |
| 2020 | NO | Särkilampi | 22 | 60 | Time-lapse | 16.6.2020 | 21.8.2020 | 66 | 6078 |
| 2020 | NO | Joutenlampi | 23 | 40 | Motion sensor | 16.6.2020 | 24.8.2020 | 69 | 563 |
| 2020 | NO | Joutenlampi | 23 | 58 | Time-lapse | 16.6.2020 | 27.6.2020 | 11 | 1016 |
| 2020 | NO | Niitty-Koivulampi | 24 | 28 | Motion sensor | 17.6.2020 | 21.8.2020 | 65 | 307 |
| 2020 | NO | Niitty-Koivulampi | 24 | 59 | Time-lapse | 17.6.2020 | 21.8.2020 | 65 | 5754 |
| 2020 | NO | Onki-Koivulampi | 25 | 81 | Motion sensor | 17.6.2020 | 21.8.2020 | 65 | 409 |
| 2020 | NO | Onki-Koivulampi | 25 | 24 | Time-lapse | 17.6.2020 | 21.8.2020 | 65 | 6061 |
| 2020 | NO | Onki-Koivulampi | 26 | 37 | Motion sensor | 17.6.2020 | 21.8.2020 | 65 | 355 |
| 2020 | NO | Onki-Koivulampi | 26 | 34 | Time-lapse | 17.6.2020 | 1.7.2020 | 14 | 1302 |
| 2020 | NO | Kultalampi | 27 | 43 | Motion sensor | 17.6.2020 | 11.7.2020 | 24 | 4970 |
| 2020 | NO | Kultalampi | 27 | 69 | Time-lapse | 17.6.2020 | 19.8.2020 | 63 | 5803 |
| 2020 | NO | Kultalampi | 28 | 78 | Motion sensor | 17.6.2020 | 19.8.2020 | 63 | 33 |
| 2020 | NO | Kultalampi | 28 | 79 | Time-lapse | 17.6.2020 | 26.7.2020 | 39 | 1855 |
|  |  |  |  |  |  |  | Total | 3417 | 159575 |

Details of game cameras deployed for taiga bean goose monitoring in 2020. L = Lapland, NK = North Karelia, NO = Northern Ostrobothnia.

**Table S2. Details of game cameras deployed in 2021.**

| Year | Province | Site | Pair | Camera | Trigger | Start date | End date | Days | Images |
| --- | --- | --- | --- | --- | --- | --- | --- | --- | --- |
| 2021 | L | Joutsenaapa | 1 | 33 | Motion sensor | 2.6.2021 | 14.9.2021 | 104 | 5790 |
| 2021 | L | Joutsenaapa | 1 | 75 | Time-lapse | 2.6.2021 | 6.7.2021 | 34 | 3168 |
| 2021 | L | Kaita-Aapa | 2 | 25 | Motion sensor | 2.6.2021 | 26.8.2021 | 85 | 292 |
| 2021 | L | Kaita-Aapa | 2 | 83 | Time-lapse | 2.6.2021 | 29.7.2021 | 56 | 5242 |
| 2021 | L | Kaita-Aapa | 3 | 48 | Motion sensor | 2.6.2021 | 14.9.2021 | 104 | 4166 |
| 2021 | L | Kaita-Aapa | 3 | 72 | Time-lapse | 2.6.2021 | 14.9.2021 | 104 | 9574 |
| 2021 | L | Sourulampi | 4 | 77 | Motion sensor | 1.6.2021 | 7.8.2021 | 67 | 1669 |
| 2021 | L | Sourulampi | 4 | 51 | Time-lapse | 1.6.2021 | 25.8.2021 | 85 | 6630 |
| 2021 | L | Suikeloaapa | 5 | 53 | Motion sensor | 1.6.2021 | 5.8.2021 | 65 | 5719 |
| 2021 | L | Suikeloaapa | 5 | 52 | Time-lapse | 1.6.2021 | 15.9.2021 | 106 | 662 |
| 2021 | L | Malja-Aapa | 6 | 85 | Motion sensor | 1.6.2021 | 30.6.2021 | 29 | 501 |
| 2021 | L | Malja-Aapa | 6 | 70 | Time-lapse | 1.6.2021 | 8.8.2021 | 68 | 5387 |
| 2021 | L | Malja-Aapa | 7 | 54 | Motion sensor | 1.6.2021 | 15.9.2021 | 106 | 284 |
| 2021 | L | Malja-Aapa | 7 | 76 | Time-lapse | 1.6.2021 | 17.7.2021 | 46 | 4256 |
| 2021 | NK | Lehmilampi | 8 | 16 | Motion sensor | 18.5.2021 | 29.6.2021 | 42 | 2629 |
| 2021 | NK | Lehmilampi | 8 | 35 | Time-lapse | 18.5.2021 | 17.8.2021 | 91 | 8447 |
| 2021 | NK | Tavilampi | 9 | 6 | Motion sensor | 20.5.2021 | 23.8.2021 | 95 | 1736 |
| 2021 | NK | Tavilampi | 9 | 5 | Time-lapse | 20.5.2021 | 23.8.2021 | 95 | 8851 |
| 2021 | NK | Teerilampi | 10 | 45 | Motion sensor | 20.5.2021 | 22.8.2021 | 94 | 3250 |
| 2021 | NK | Teerilampi | 10 | 56 | Time-lapse | 20.5.2021 | 15.7.2021 | 56 | 5197 |
| 2021 | NK | Rääkälelammit | 11 | 22 | Motion sensor | 21.5.2021 | 1.8.2021 | 72 | 7206 |
| 2021 | NK | Rääkälelammit | 11 | 11 | Time-lapse | 21.5.2021 | 24.8.2021 | 95 | 8839 |
| 2021 | NK | Rääkälelammit | 12 | 38 | Motion sensor | 21.5.2021 | 24.8.2021 | 95 | 1505 |
| 2021 | NK | Rääkälelammit | 12 | 30 | Time-lapse | 23.6.2021 | 24.8.2021 | 62 | 5649 |
| 2021 | NK | Pirttilammit | 13 | 32 | Motion sensor | 21.5.2021 | 24.8.2021 | 95 | 5206 |
| 2021 | NK | Pirttilammit | 13 | 36 | Time-lapse | 21.5.2021 | 22.8.2021 | 93 | 8501 |
| 2021 | NK | Pirttilammit | 14 | 49 | Motion sensor | 21.5.2021 | 24.8.2021 | 95 | 260 |
| 2021 | NK | Pirttilammit | 14 | 7 | Time-lapse | 21.5.2021 | 24.8.2021 | 95 | 8473 |
| 2021 | NO | Pieni Mustikkalampi | 15 | 55 | Motion sensor | 16.6.2021 | 17.8.2021 | 62 | 245 |
| 2021 | NO | Pieni Mustikkalampi | 15 | 82 | Time-lapse | 16.6.2021 | 17.8.2021 | 62 | 5824 |
| 2021 | NO | Pieni Mustikkalampi | 16 | 40 | Motion sensor | 16.6.2021 | 17.8.2021 | 62 | 1229 |
| 2021 | NO | Pieni Mustikkalampi | 16 | 79 | Time-lapse | 16.6.2021 | 17.8.2021 | 62 | 5759 |
| 2021 | NO | Mustikkalampi | 17 | 43 | Motion sensor | 16.6.2021 | 29.6.2021 | 13 | 3406 |
| 2021 | NO | Mustikkalampi | 17 | 17 | Time-lapse | 16.6.2021 | 19.6.2021 | 3 | 302 |
| 2021 | NO | Mustikkalampi | 18 | 74 | Motion sensor | 16.6.2021 | 17.8.2021 | 62 | 392 |
| 2021 | NO | Mustikkalampi | 18 | 87 | Time-lapse | 16.6.2021 | 20.7.2021 | 34 | 2995 |
| 2021 | NO | Kalhulampi | 19 | 15 | Motion sensor | 16.6.2021 | 16.8.2021 | 61 | 421 |
| 2021 | NO | Kalhulampi | 19 | 66 | Time-lapse | 16.6.2021 | 10.7.2021 | 24 | 2074 |
| 2021 | NO | Kalhulampi | 20 | 13 | Motion sensor | 16.6.2021 | 16.8.2021 | 61 | 1050 |
| 2021 | NO | Kalhulampi | 20 | 34 | Time-lapse | NA^a^ | NA^a^ | 0^a^ | 0^a^ |
| 2021 | NO | Särkilampi | 21 | 28 | Motion sensor | 16.6.2021 | 3.7.2021 | 17 | 895 |
| 2021 | NO | Särkilampi | 21 | 60 | Time-lapse | 16.6.2021 | 28.7.2021 | 42 | 3917 |
| 2021 | NO | Särkilampi | 22 | 39 | Motion sensor | 16.6.2021 | 17.8.2021 | 62 | 621 |
| 2021 | NO | Särkilampi | 22 | 59 | Time-lapse | 16.6.2021 | 21.7.2021 | 35 | 3290 |
| 2021 | NO | Joutenlampi | 23 | 78 | Motion sensor | 9.6.2021 | 16.8.2021 | 68 | 482 |
| 2021 | NO | Joutenlampi | 23 | 58 | Time-lapse | 9.6.2021 | 16.8.2021 | 68 | 6154 |
| 2021 | NO | Niitty-Koivulampi | 24 | 50 | Motion sensor | 14.6.2021 | 25.6.2021 | 11 | 67 |
| 2021 | NO | Niitty-Koivulampi | 24 | 84 | Time-lapse | 14.6.2021 | 15.7.2021 | 31 | 2929 |
| 2021 | NO | Onki-Koivulampi | 25 | 80 | Motion sensor | 14.6.2021 | 16.8.2021 | 63 | 397 |
| 2021 | NO | Onki-Koivulampi | 25 | 69 | Time-lapse | 14.6.2021 | 16.8.2021 | 63 | 5756 |
| 2021 | NO | Onki-Koivulampi | 26 | 37 | Motion sensor | 14.6.2021 | 16.8.2021 | 63 | 363 |
| 2021 | NO | Onki-Koivulampi | 26 | 24 | Time-lapse | 14.6.2021 | 4.7.2021 | 20 | 1835 |
| 2021 | NO | Kultalampi | 27 | 57 | Motion sensor | 17.6.2021 | 17.8.2021 | 61 | 663 |
| 2021 | NO | Kultalampi | 27 | 12 | Time-lapse | NA^a^ | NA^a^ | 0^a^ | 0^a^ |
|  |  |  |  |  |  |  | Total | 3345 | 180155 |

Details of game cameras deployed for taiga bean goose monitoring in 2021. L = Lapland, NK = North Karelia, NO = Northern Ostrobothnia.

^a^ camera failed to start
